# Depth-Profiling XPS Study of Oxygen Diffusion and Reduction During Low-Temperature Activation of Ti-Co-Ce Getter Films

**DOI:** 10.3390/ma19071379

**Published:** 2026-03-31

**Authors:** Siwei Tang, Yuhua Xiong, Huating Wu

**Affiliations:** 1 State Key Laboratory of Advanced Materials for Intelligent Sensing, GRINM Group Co., Ltd., Beijing 100088, China; 15334570574@163.com; 2 GRIMAT Engineering Institute Co., Ltd., Beijing 101407, China; wuhuating@grinm.com; 3 General Research Institute for Nonferrous Metals, Beijing 100088, China

**Keywords:** Ti-Co-Ce, non-evaporable getter films, low-temperature activation, oxygen diffusion, depth-profiling XPS, activation mechanism

## Abstract

In this study, Ti-Co-Ce getter films were deposited via magnetron sputtering to investigate their activation mechanism—the thermal removal of surface passivation layers to restore gas sorption capability. The morphology before and after film activation was characterized using scanning electron microscopy (SEM) and atomic force microscopy (AFM). The oxygen content on the film surface before and after activation was measured using an energy-dispersive X-ray spectrometer (EDS), and gas desorption during activation was monitored with a quadrupole mass spectrometer (QMS). The combined results confirmed the absence of O_2_ desorption during activation, suggesting oxygen migration into the film bulk. Crucially, in situ X-ray photoelectron spectroscopy (XPS) combined with controlled Ar^+^ ion sputtering depth profiling (0–30 nm) was employed to directly probe the chemical-state evolution within the thin film before and after thermal activation at 400 °C, thereby providing direct evidence of the activation dynamics. The data reveal that within the 0–10 nm near-surface region, a strong oxygen chemical potential gradient drives rapid oxide reduction and inward migration of lattice oxygen. At depths of 20–30 nm, moderate reduction coupled with oxygen enrichment induces phase separation, while around 30 nm, a dynamic equilibrium between oxygen inflow and outflow is established. These findings provide a theoretical basis for optimizing activation processes and guiding the development of low-temperature getter materials. This work is particularly relevant for MEMS, vacuum electronics, and other applications with stringent thermal budgets, expanding the design possibilities for heat-sensitive device integration.

## 1. Introduction

Non-evaporable getter (NEG) films are critical for ensuring the long-term stable operation of particle accelerators, synchrotron radiation facilities, and various sealed vacuum devices [1,2,3,4]. Their ability to capture active gases through surface adsorption and bulk diffusion plays a crucial role in achieving and maintaining high vacuum conditions. As vacuum electronic devices continue to evolve toward miniaturization and micro-miniaturization, there is a growing demand for further reductions in activation temperature and improvements in gas absorption performance for getter films. This trend drives the research focus toward developing getter films with lower activation temperatures and enhanced performance.

Currently, the widely used commercial and research-grade getter film material systems include Ti-Zr-V, Zr-Co-Ce, and Ti-Zr-Hf-V. These material systems generally exhibit excellent gas absorption properties and can be activated at relatively low temperatures. However, for certain heat-sensitive components, the activation temperature of existing systems may still be excessively high, posing a risk of thermal damage. To reduce the risk of thermal damage to vacuum components and to enhance the application compatibility of getter films, further research is needed on novel getter film material systems that enable activation at lower temperatures [5,6] while delivering superior gas absorption performance. To overcome these technical bottlenecks, the research focus is gradually shifting from material screening to a deeper understanding of the activation mechanisms at the microscopic level. This shift aims to establish a solid theoretical foundation for the rational design and development of next-generation high-performance getter film materials capable of low-temperature activation.

After exposure to the atmosphere, the highly reactive surface of the prepared film rapidly interacts with gas molecules in the environment (such as H_2_O, CO_2_, and hydrocarbons), forming a dense passivation layer [7,8,9]. Activation refers to the process of removing the passivation layer by desorbing physisorbed and chemisorbed species and reducing the native surface oxide layer through physical or chemical means. This exposes a “fresh” active surface and restores the gas sorption activity of the getter film. The most common activation method involves heating the material in a vacuum or inert atmosphere and maintaining it at that temperature for a specified period [10,11,12,13]. While it is widely recognized in the academic community that the activation process involves oxide reduction and oxygen diffusion into the bulk phase, direct experimental observations of the dynamic evolution of chemical states, particularly beneath the surface layer, remain quite limited [13,14,15,16]. For example, F. Sutara et al. [16] investigated changes in the Ti-Zr-V surface during thermal activation using XPS and ESD testing methods. Sihui Wang et al. [17] employed in situ synchrotron radiation X-ray photoelectron spectroscopy to quantitatively analyze the chemical state evolution of the Ti-Zr-V thin film surface. They found that active metallic elements gradually reduced from their initial highly oxidized state to low-valent oxides, ultimately reverting to a metallic state. Previous studies employing surface-sensitive techniques such as XPS have primarily focused on compositional changes at the topmost layer, resulting in an insufficient understanding of subsurface oxygen migration pathways. This study addresses this gap by combining in situ XPS with controlled Ar^+^ depth profiling to directly probe chemical state evolution from the surface down to approximately 30 nm. This approach enables, for the first time in Ti-Co-Ce getter films, a comprehensive mapping of the depth-dependent oxygen reduction and diffusion dynamics during thermal activation. The findings provide direct experimental evidence for the oxygen migration pathway—from surface oxide reduction to inward diffusion and eventual dynamic equilibrium—thereby advancing the fundamental understanding of low-temperature activation mechanisms beyond the existing literature that has largely focused on surface phenomena.

In recent years, our laboratory has successfully developed a novel ternary Ti-Co-Ce getter thin-film material [18]. Under identical preparation and testing conditions, the Ti-Co-Ce bulk getter exhibits superior hydrogen-absorption performance at room temperature compared with Zr-Co-Ce (Zr_76.2_Co_20.7_Ce_3.1_ (at.%)) [19,20,21]. Based on this, this study will focus on the Ti-Co-Ce getter film as its core research subject, with an emphasis on elucidating the oxygen reduction and diffusion mechanisms during the activation process of the Ti-Co-Ce film. We employed a comprehensive suite of techniques, including scanning electron microscopy (SEM), atomic force microscopy (AFM), energy-dispersive X-ray spectroscopy (EDS), and quadrupole mass spectrometry (QMS). Particularly, by integrating in situ XPS with depth profiling, we comprehensively revealed the activation process from the surface down to approximately 30 nanometers in depth. Through dynamic changes in the valence states and distribution of key elements such as Ti and O during activation, direct evidence for oxygen reduction is provided, the spatial migration pathway of oxygen is elucidated, and the low-temperature activation behavior of Ti-Co-Ce is clarified.

## 2. Experimental

### 2.1. Sample Preparation

In this study, a Ti_85.2_Co_13.0_Ce_1.8_ (at. %) alloy target is self-made and has a purity greater than 99.5%. The film was deposited by DC magnetron sputtering onto p-type single-crystal Si (100) substrates. Prior to deposition, the base pressure of the coating chamber was pumped down to better than 4 × 10^−4^ Pa. High-purity argon was introduced as the sputtering gas at a flow rate of 60 sccm. The sputtering pressure was maintained at 4.0 Pa with a power density of 1 W/cm^2^. The distance between the target and the substrate was set to 8 cm, and the substrate rotation speed was 5 rpm. Under these conditions, a film with a thickness of approximately 1.5 μm was obtained.

### 2.2. Activation Process

The activation process is defined as a thermal treatment conducted in a vacuum or inert atmosphere, aimed at removing the surface passivation layer composed of oxides, hydroxides, carbonates, and adsorbed gaseous species, thereby exposing a fresh metallic active surface and restoring the film’s gas sorption capacity. This regeneration step is indispensable for the practical application of getter films. Samples were heated in a vacuum chamber with pressure maintained below 10^−6^ Pa. The film was heated to specified temperatures (300 °C, 350 °C, 400 °C) and held for half an hour. These temperatures were selected based on our preliminary experiments and literature reports on similar getter systems, covering the range from below the typical activation threshold (300 °C) to the empirically determined effective activation temperature (400 °C) for Ti-Co-Ce films, allowing systematic observation of the temperature-dependent activation behavior.

### 2.3. Characterization

A JEOL JSM-7900F scanning electron microscope (SEM) equipped with an energy-dispersive X-ray spectrometer (EDS) (JEOL Ltd., Akishima, Tokyo, Japan) was used to characterize the surface and cross-sectional morphology, grain size, and elemental composition of the getter film were characterized before and after activation. SEM observations were performed at an acceleration voltage of 5 kV and a working distance of 8.3 mm. The evaluation of surface roughness was performed under ambient conditions using a BRUKER Dimension Icon atomic force microscope (AFM) (Bruker Corporation, Billerica, MA, USA) in non-contact tapping mode. AFM scanning area was 5 μm × 5 μm, with a scanning rate of 0.8 Hz.

The hydrogen absorption performance of Ti-Co-Ce getter films activated at 400 °C was tested using the constant-pressure method. The measurement principle of this method is detailed in ASTM F798-97(2002) [22]. The Ti-Co-Ce getter films were activated under vacuum conditions, and after cooling to room temperature, their hydrogen absorption performance was tested. During the test, a constant H_2_ pressure of 4.0 × 10^−4^ Pa above the getter, *P*_g_ was maintained, and the change in the inlet chamber pressure *P*_m_ over time was recorded for 2 h. The pumping speed and sorption capacity were calculated according to the corresponding formula.

Gas desorption during the activation process was monitored using a Pufa QMG 250-100 quadrupole mass spectrometer (QMS) (Pfeiffer Vacuum GmbH, Asslar, Germany). The test was conducted under a vacuum exceeding 10^−6^ Pa, with the sample heated according to a preset program: temperature ranges of 25–100 °C, 100–200 °C, 200–300 °C, and 300–400 °C, maintaining each temperature point at 100, 200, 300, and 400 °C for 15 min.

In situ XPS and depth-profiling analyses were performed on a Thermo Fisher Scientific ESCALAB 250Xi X-ray photoelectron spectrometer (Thermo Fisher Scientific, Waltham, MA, USA) to investigate the evolution of elemental valence states and distribution in Ti-Co-Ce getter films during activation [23,24,25,26]. Measurements were conducted in an analysis chamber under a vacuum of 8 × 10^−10^ Pa, using monochromatic Al Kα radiation (hv = 1486.8 eV) as the excitation source with an operating voltage of 12.5 kV and a filament current of 16 mA. The X-ray spot size was approximately 650 μm, defining the analyzed area. Spectra were collected with a pass energy of 40 eV and a step size of 0.1 eV, and signal accumulation over 5–10 cycles was applied to enhance the signal-to-noise ratio. All binding energies were charge-corrected with reference to the C 1s peak at 284.60 eV. Spectral deconvolution was performed using a curve-fitting program based on iterative least-squares methods [27]. For O 1s spectra, two primary peaks were defined within the binding energy ranges of 529.5–530.5 eV (lattice oxygen) and 530.5–531.5 eV (surface hydroxyl groups). During fitting, the full width at half maximum (FWHM) of all components was constrained to similar values (within ±0.2 eV) to ensure physically meaningful results, and the fitting was optimized through iterative refinement until convergence with minimal residuals.

Prior to analysis, the sample was degassed at 100 °C for 12 h. The experimental procedure consisted of the following steps. First, XPS data were collected from the film surface at room temperature. Depth profiling was then performed via Ar^+^ ion sputtering, with spectra acquired at sputtered depths of approximately 10, 20, and 30 nm. Ar^+^ sputtering was conducted with an ion beam energy of 2 keV, a filament current of 16 mA, and a rastered area of approximately 2 mm × 2 mm, resulting in a current density of approximately 2 μA/mm^2^. The sputtering rate was calibrated to be approximately 2 nm/min under these conditions using a standard SiO_2_/Si reference sample. To obtain depth-resolved information, sequential sputtering was conducted with a sputtering duration of 77 s per cycle, corresponding to an estimated depth increment of approximately 2.6 nm per cycle. XPS spectra were acquired after reaching cumulative depths of approximately 10 nm, 20 nm, and 30 nm (achieved after 4, 8, and 12 sputtering cycles, respectively). Subsequently, the sample was heated to 400 °C under vacuum and held for 0.5 h, after which surface spectra were collected at this temperature, followed by repetition of the aforementioned sputtering and measurement sequence. This procedure allowed us to obtain information on the evolution of chemical states and distribution of Ti, Co, Ce, O, C, and N along the depth direction during the activation process.

## 3. Results and Discussion

### 3.1. Microstructural Characterization

**Figure 1:**
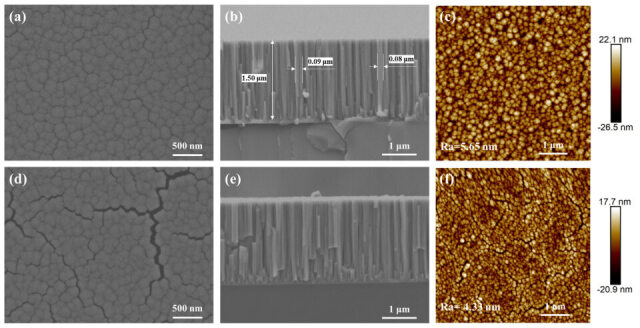
Morphological and structural characterization of the Ti-Co-Ce getter film before and after thermal activation. The as-deposited film: (**a**) surface, (**b**) cross-sectional, and (**c**) AFM images. The film after activation at 350 °C: (**d**) surface, (**e**) cross-sectional, and (**f**) AFM images.

Figure 1a presents the surface morphology of a Ti-Co-Ce getter film deposited by magnetron sputtering. The surface is composed of cauliflower-like clusters with a range of pore sizes and no specific geometric shape. Figure 1b shows the fresh cross-sectional morphology of the deposited film, exhibiting a relatively uniform and continuous columnar crystal structure consistent with the typical characteristics of the Thornton growth model [13,26]. The columnar crystals have diameters in the range of 30–90 nm; they are nanocolumnar crystals. The boundaries between the crystals are well-defined and distinct, with each crystal separated from the others. The cross-sectional SEM image shows a film thickness of approximately 1.50 μm. The arithmetic mean roughness Ra of the film surface, measured from the three-dimensional topography image in Figure 1c, is approximately 5.65 nm. After activating the film at 350 °C, it was similarly examined using SEM. Figure 1d shows the surface morphology of the activated film, which exhibits significant changes compared to the as-deposited state. The high-temperature treatment induces pronounced sintering effects, resulting in the formation of a network of wide and deep cracks on the surface. Figure 1e shows the fresh cross-sectional morphology of the activated film. It is evident that the columnar crystal structure largely retains its original orientation, but the gaps between the columnar crystals have widened, with signs of grain boundary separation appearing in some areas. The expansion of these gaps provides additional pathways for gas adsorption and reflects the regulatory effect of activation treatment on the microstructure within the film. Figure 1f shows the AFM test results of the activated film. After activation, the arithmetic mean roughness of the film surface decreased to Ra = 4.33 nm.

**Figure 2:**
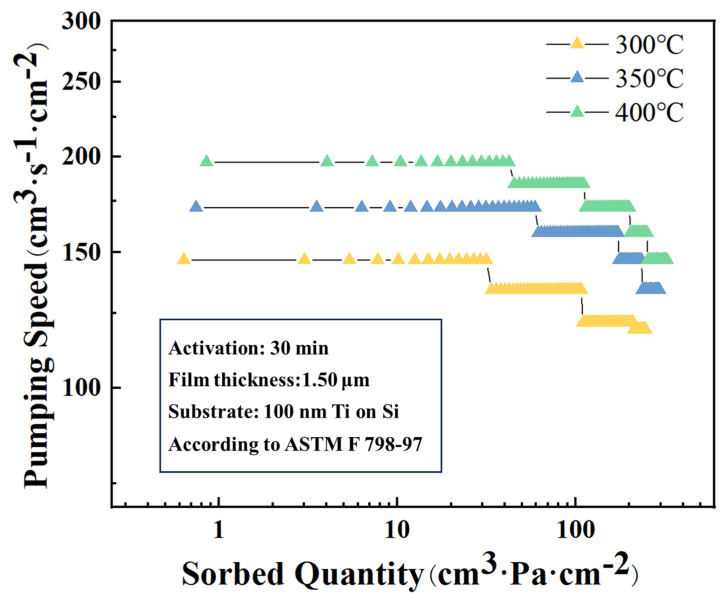
Hydrogen absorption performance plot of Ti-Co-Ce getter films under different activation temperatures.

The hydrogen adsorption performance of Ti-Co-Ce getter films activated at 300 °C, 350 °C, and 400 °C was evaluated, as summarized in Figure 2. The results indicate that the hydrogen adsorption capacity increases with higher activation temperature. At lower activation temperatures, adsorbed species such as moisture and CO_2_ are not fully desorbed, and the surface oxide layer remains incompletely reduced. As a result, active metallic sites are insufficiently exposed, limiting the availability of effective adsorption sites. With increasing activation temperature, thermal activation promotes both the decomposition of the surface oxide layer and the complete removal of impurities, leading to a greater number of exposed active metal sites and consequently enhanced gas sorption performance of the getter film. Compared with literature data, the hydrogen absorption capacity of Ti-Co-Ce films activated at 400 °C (reaching approximately 4.5 × 10^−2^ Pa·m^3^/mg after 2 h) is higher than that of Zr-Co-Ce films reported by Xiong et al. [18] (approximately 3.8 × 10^−2^ Pa·m^3^/mg under similar conditions), demonstrating the competitive performance of the Ti-Co-Ce system. This comparative analysis demonstrates that the Ti-Co-Ce system achieves effective activation at 400 °C with competitive gas sorption performance, confirming its potential as a low-temperature getter material.

### 3.2. Oxygen Behavior During Activation

**Figure 3:**
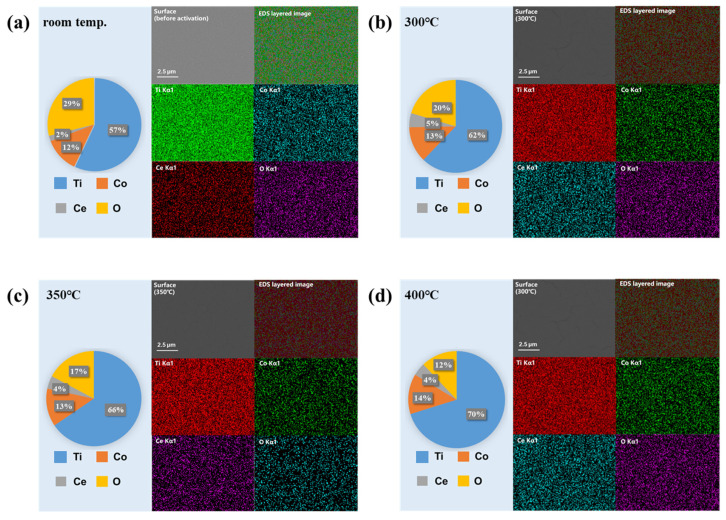
EDS mapping results of Ti-Co-Ce getter film: (**a**) before activation; (**b**) after activation at 300 °C; (**c**) after activation at 350 °C; (**d**) after activation at 400 °C.

Figure 3 shows the EDS surface scanning analysis results of Ti-Co-Ce getter films in their as-deposited state and after activation treatment at different temperatures (300 °C, 350 °C, 400 °C). In the deactivated state, the exposed film interacts with atmospheric gases, leading to the formation of a passivation layer on its surface and the adsorption of water molecules, carbon dioxide, and other oxygen-containing compounds. Consequently, EDS spectra reveal a relatively high proportion of oxygen elements. Following activation treatment, heating promotes the reduction reaction of surface and shallow-layer oxides on the film, while simultaneously desorbing adsorbed oxide compounds, thereby significantly reducing surface oxygen content. As the activation temperature gradually increases, the oxide layer decomposes more thoroughly, and oxides with stronger adsorption capabilities are removed more effectively, leading to a further reduction in residual oxygen content on the surface. EDS quantitative analysis indicates that the surface oxygen content decreases significantly from 29.0 ± 1.2 at.% (as-deposited) to 20.4 ± 0.9 at.%, 17.1 ± 0.8 at.%, and 12.4 ± 0.7 at.% after activation at 300 °C, 350 °C, and 400 °C, respectively (mean ± SD, n = 5 area scans per condition). This progressive reduction confirms the temperature-dependent efficiency of oxide removal. EDS analysis indicates that the oxygen content in the activated film is significantly lower than that in the as-deposited state, and the decrease in oxygen content progressively increases with rising activation temperature.

**Figure 4:**
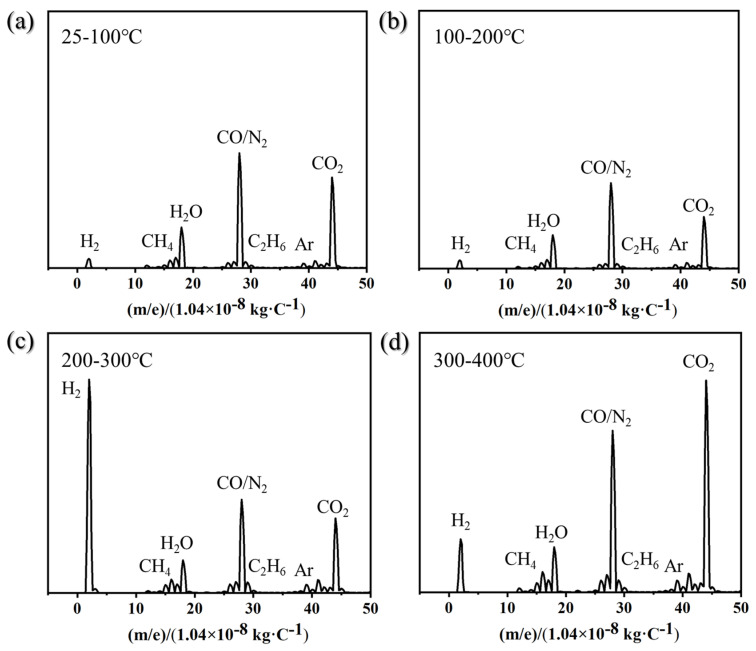
Residual gas mass spectra during the activation process of Ti-Co-Ce getter film: (**a**) 25–100 °C; (**b**) 100–200 °C; (**c**) 200–300 °C; (**d**) 300–400 °C.

The activation process of getter films essentially involves heating in a vacuum environment to promote the gradual desorption of various adsorbed gas molecules (such as H_2_O, CO_2_, CO, N_2_, H_2_, CH_4_, C_2_H_6_, etc.), which gradually desorb and detach along the temperature gradient. Simultaneously, surface oxides undergo thermal reduction, and the passivation layer decomposes, ultimately exposing a clean, highly active fresh metal surface. This lays the foundation for effectively capturing residual gases within subsequent devices. Figure 4 shows the real-time monitoring results of gas components released during the activation process of Ti-Co-Ce getter films using a quadrupole mass spectrometer (QMS). The figure indicates that the residual gases released during activation primarily consist of H_2_, CH_4_, H_2_O, CO/N_2_, C_2_H_6_, Ar, and CO_2_. Among these, CO and N_2_ are difficult to distinguish in single-charge ion detection mode due to their identical molecular weights, and are therefore identified as mixed components. The corresponding mass-to-charge ratios (×1.04 × 10^−8^ m/e) for these gases are 2, 16, 18, 28, 30, 40, and 44 kg/C.

Analysis of mass spectrometry trends reveals that the relative abundances of different gas components exhibit significant variations with changes in activation temperature. Within the 25–100 °C temperature range, desorbed gases primarily consist of physically adsorbed or weakly chemically adsorbed components, with H_2_O molecules predominantly desorbing during this stage. In the 100–200 °C range, CO/N_2_ peaks show a slight decrease. From 200–300 °C, H_2_ becomes the predominant desorbed gas. Entering the 300–400 °C range, the H_2_ signal remains at a relatively high level but shows a slight decline, while the desorption peak for CO_2_ significantly increases. Overall, the 25–200 °C range can be regarded as the desorption phase dominated by physical adsorption. This stage primarily removes impurities such as adsorbed water vapor, air components, and trace hydrocarbons from the material surface. It serves as a critical step for achieving preliminary surface purification, thereby reducing interference with subsequent high-temperature activation processes. Within the 200–400 °C range, the process primarily involves the desorption of chemically adsorbed gases, accompanied by the reduction of the oxide layer and the exposure of active sites. This stage constitutes the core process through which the adsorbent acquires its actual gas adsorption capacity.

It is noteworthy that no distinct O_2_ desorption signal was detected throughout the entire activation process. However, combined with the aforementioned EDS analysis results, it is evident that the oxygen content on the film surface decreased significantly after activation. By integrating QMS and EDS data, it can be inferred that oxygen in metal oxides does not release as oxygen gas during heating. Instead, it primarily migrates from the surface into the bulk phase of the film through diffusion. This behavior aligns with the currently accepted industry understanding of oxygen dynamics during activation processes.

### 3.3. XPS Analysis of Oxygen Behavior During Activation

#### 3.3.1. Surface Survey and Elemental Quantification

**Figure 5:**
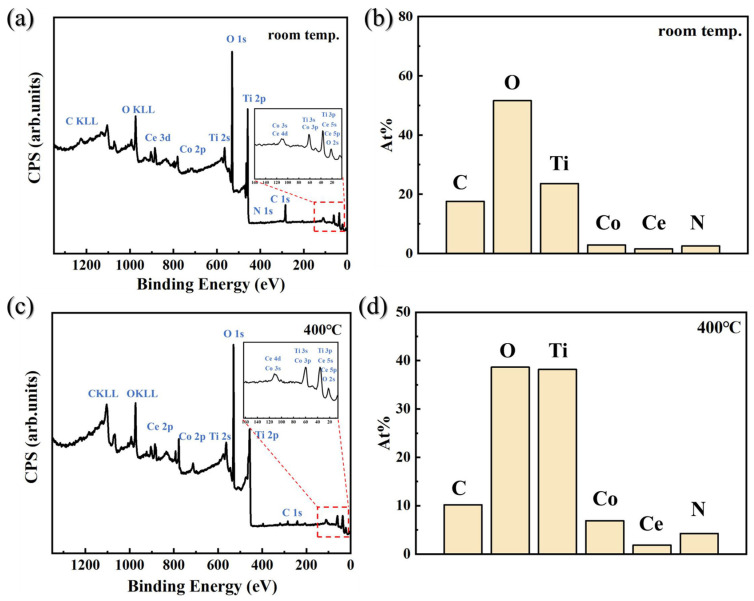
Ti-Co-Ce getter film as-deposited surface: (**a**) XPS survey spectrum; (**b**) elemental content distribution. Surface activated at 400 °C: (**c**) XPS survey spectrum; (**d**) elemental content distribution.

The chemical states and concentration distributions of each element in Ti-Co-Ce getter films were investigated before and after activation through in situ XPS characterization. Figure 5a shows the full XPS spectrum of the thin film surface at room temperature. The results indicate that the film surface primarily consists of elements such as Ti, Co, Ce, C, O, and N. The relative abundances of each element are shown in Figure 5b. The O 1s peak is relatively strong, primarily originating from surface-adsorbed oxygen-containing species and the oxide layer; the C signal mainly stems from system contamination or surface adsorption. The relatively weak signals for Ce and Co are due to their lower concentrations. Subsequently, the sample was heated to 400 °C and held at this temperature for 0.5 h for activation treatment. XPS testing was performed again on the treated surface, yielding the spectrum shown in Figure 5c. Comparing the XPS data before and after activation reveals significant changes in the relative intensities of each element, as shown in Figure 5d. Quantitative analysis of the survey spectra indicates a strong increase (over 60%) in Ti 2p intensity and a significant decrease (approximately 25%) in O 1s intensity after activation. This trend indicates that the activation process effectively removed surface carbon-oxygen contamination and promoted the exposure of metallic elements. Based on the preliminary analysis results, oxygen does not desorb in gaseous form during the activation process but migrates from the surface layer by diffusing into the bulk phase of the film. To investigate the oxygen migration and reduction mechanisms in this process, deep etching experiments were conducted on the films before and after activation. High-resolution XPS spectra for Ti 2p and O 1s were obtained at the surface and at depths of 10 nm, 20 nm, and 30 nm, respectively. The chemical state information evolving with depth was systematically analyzed to reveal the dynamic behavior of oxygen reduction and diffusion in Ti-Co-Ce getter films during heat treatment.

#### 3.3.2. Ti 2p Depth Profiling

Titanium, as one of the core active components in Ti-Co-Ce getter films, possesses multiple variable chemical oxidation states (such as Ti(IV), Ti(III), Ti(II), and Ti^0^). Its XPS spectrum reflects variations in oxygen environments at different depths, thereby providing crucial insights into the reduction and diffusion behavior of oxygen during the film activation process.

**Figure 6:**
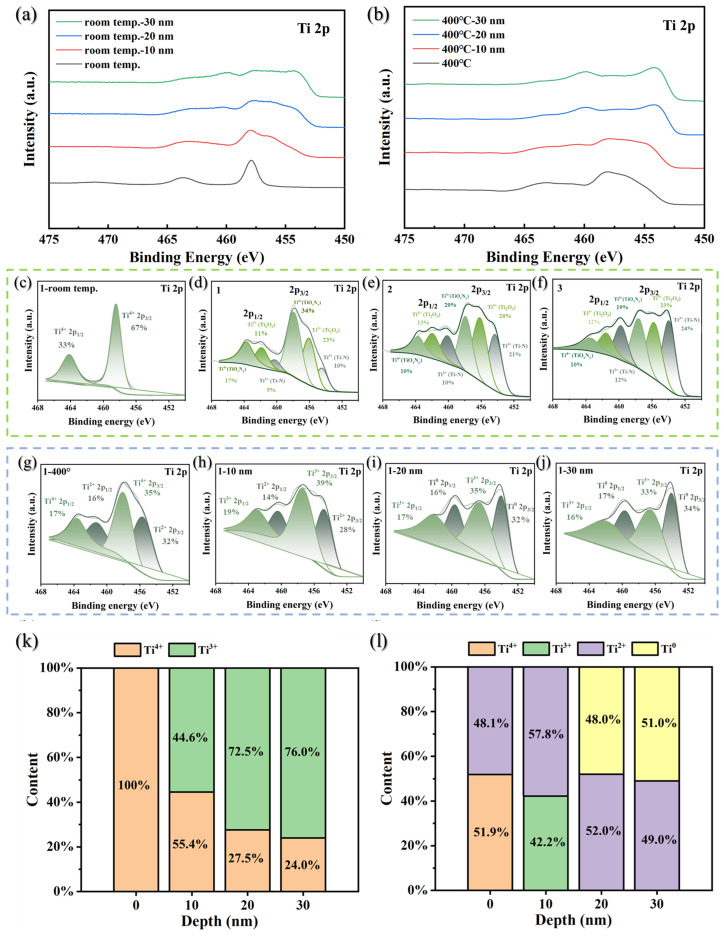
Ti 2p characteristic peaks of Ti-Co-Ce getter film scanned by XPS: (**a**) as-deposited state; (**b**) after activation at 400 °C. Deconfiguration of Ti 2p characteristic peaks in the as-deposited state: (**c**) surface; (**d**) 10 nm; (**e**) 20 nm; (**f**) 30 nm. Ti 2p characteristic peak deconvolution after 400 °C activation: (**g**) surface; (**h**) 10 nm; (**i**) 20 nm; (**j**) 30 nm. Distribution of Ti in various valence states: (**k**) as-deposited state; (**l**) after 400 °C activation.

Figure 6a,b show the high-resolution XPS spectra of the Ti 2p orbitals at different depths for Ti-Co-Ce getter films in their as-deposited state and after activation treatment at 400 °C, respectively.

As shown in Figure 6a, the Ti 2p spectrum of the film surface exhibits a typical spin–orbit split double-peak structure at room temperature. As the etching depth progressively extends into the film interior, this double peak gradually broadens, and its peak sharpness decreases, indicating that the chemical environment of Ti changes with depth. After activation treatment involving 0.5 h of isothermal heating at 400 °C, the surface Ti 2p XPS spectrum exhibited significant changes.

As shown in Figure 6b, compared to the as-deposited state, the intensity in the valley region between the Ti 2p double peaks increased on the activated film surface. The double peak profiles became smoother, with a faint peak emerging between them, preliminarily indicating the initiation of Ti reduction. Further layer-by-layer etching (10 nm, 20 nm, 30 nm) of the activated samples revealed that compared to the in situ deposited state, the activated Ti 2p spectrum gradually evolved from its initial double-peak structure into four distinct peaks, with two new minor peaks emerged on top of the original double peaks. And their intensities progressively increased with etching depth. This indicates that the low-valent Ti components within the film became increasingly prominent with increasing depth.

Ti(IV) is the fully oxidized state, typically tightly bound to lattice oxygen, while Ti(III) and Ti(II) are intermediate states resulting from the reduction of Ti(IV), and their presence indicates the occurrence of reduction reactions. The Ti 2p spectra of the deposited state and the film activated at 400 °C were subjected to peak resolution.

Figure 6c–f shows the high-resolution Ti 2p XPS peak fitting spectra of the film surface at room temperature and after etching to depths of 10 nm, 20 nm, and 30 nm. The Ti 2p core-level splits into two characteristic peaks, 2p3/2 and 2p1/2, due to spin–orbit coupling [27,28]. The 2p3/2 peak is the dominant one and is commonly used for chemical state analysis. On the deposited film surface, the main Ti 2p3/2 peak is located at a binding energy of 458.4 eV (FWHM = 1.5 eV), indicating that Ti primarily exists in the stable TiO_2_ (Ti(IV)) form. As the etching depth increases to 10–30 nm, two new peaks appear at approximately 454.0 eV and 456.0 eV. Referencing the XPS standard database, these peaks can be attributed to Ti(III) species. Thus, Ti in the deposited film primarily exists as Ti(IV) and Ti(III).

Figure 6g–j shows the Ti 2p peak spectra at the surface and different depths of the film after activation treatment at 400 °C. The heat treatment significantly altered the distribution of Ti’s chemical states. On the film surface, the Ti 2p spectrum can be fitted to two peaks: one at 455.6 eV (FWHM = 2.8 eV, corresponding to Ti(II)) and another at 458.0 eV (FWHM = 2.6 eV, corresponding to Ti(IV)), indicating the formation of a mixed state where Ti(II) and Ti(IV) coexist on the surface. At a depth of approximately 10 nm, the spectral lines exhibit significant broadening, yielding two fitted peaks at 454.7 eV (Ti(III)) and 457.3 eV (Ti(II)). The Ti(IV) peak disappears, indicating the coexistence of Ti(III) and Ti(II). Further analysis in the 20–30 nm region reveals that the Ti 2p spectrum decomposes into two peaks at 454.0 eV (Ti^0^) and 456.6 eV (Ti(III)), indicating the presence of a mixed form of Ti^0^ and Ti(III) in this region.

Quantitative analysis of the peak areas was used to determine the distribution of Ti valence states with depth, as summarized in Figure 6k,l.

Figure 6k shows that the deposited film surface consists almost entirely of Ti(IV), indicating that the surface layer has been fully oxidized to form TiO_2_. As the film was etched layer by layer toward the interior (10 nm, 20 nm, 30 nm), the proportion of Ti(III) gradually increased (45%, 73%, 76%, respectively). Meanwhile, Ti(IV) correspondingly decreased, reflecting a gradual weakening of oxidation with increasing depth.

Figure 6l indicates that after activation at 400 °C, the valence state composition of Ti undergoes significant alteration. The film surface no longer contains only Ti(IV), but instead exhibits up to 48% Ti(II). Ti(II), as an extremely unstable intermediate oxidation state, cannot persist stably in the atmospheric environment. Their significant presence points to their formation as products of reduction during the vacuum thermal activation process. Within a depth range of approximately 10 nm, the chemical composition of Ti undergoes further changes. This region is composed of 58% Ti(II) and 42% Ti(III), with Ti(IV) having completely disappeared. As the depth increases to approximately 20–30 nm, Ti^0^ begins to appear, accounting for approximately 50% of the composition, with the remainder primarily consisting of Ti(III).

Regarding the identification of Ti(II) species, it should be noted that Ti(II) compounds are generally strong reducing agents with low stability under ambient conditions. However, under ultra-high vacuum (UHV) conditions during XPS analysis and following Ar^+^ sputtering, the presence of Ti(II) has been documented in several studies on reduced titanium oxides [27,28]. The UHV environment prevents re-oxidation, and the sputtering process can induce preferential removal of oxygen, leading to the formation of lower oxidation states. Therefore, the Ti(II) component observed in our depth-profiled spectra after in situ activation at 400 °C is considered plausible within the experimental context. To further validate this assignment, we carefully re-examined our fitting parameters and confirmed that an alternative fit assigning these features solely to Ti(III) resulted in unacceptably large fitting residuals and inconsistent FWHM values. Thus, the coexistence of Ti(II) and Ti(III) best represents the gradual reduction gradient from the surface inward.

Figure 6k,l summarizes the distribution of Ti valence states at different depths before and after activation. The depth-dependent evolution of Ti oxidation states provides direct evidence for the oxygen reduction and diffusion dynamics during thermal activation.

#### 3.3.3. O 1s Depth Profiling

**Figure 7:**
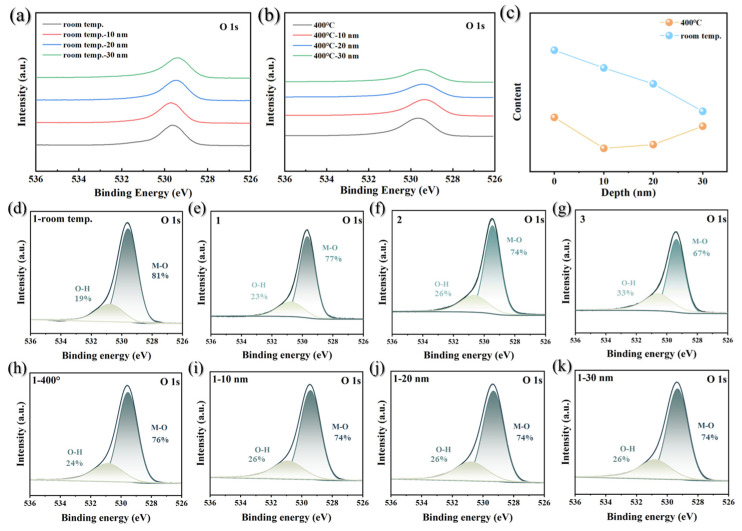
Ti-Co-Ce getter film XPS scanning O 1s characteristic peaks: (**a**) as-deposited state; (**b**) after activation at 400 °C; (**c**) lattice oxygen content variation with depth before and after activation. As-deposited O 1s characteristic peak deconvolution: (**d**) surface; (**e**) 10 nm; (**f**) 20 nm; (**g**) 30 nm. O 1s characteristic peak deconvolution after activation at 400 °C: (**h**) surface; (**i**) 10 nm; (**j**) 20 nm; (**k**) 30 nm.

To further elucidate the reduction and diffusion behavior of oxygen during the activation of Ti-Co-Ce getter films, we systematically analyzed changes in the O 1s peak before and after activation. O 1s spectra can typically be divided into two main components: lattice oxygen and OH^−^ adsorbed oxygen. Lattice oxygen exists as stable chemical bonds within the crystal structure (e.g., M-O), forming the framework of the material with high thermal stability. In contrast, OH^−^ acts as surface-adsorbed oxygen with higher reactivity, most of which can be removed during heating. This study focuses on the evolution of lattice oxygen during the activation process to elucidate the mechanisms of oxygen migration and reduction. Figure 7a,b show the high-resolution XPS O 1s spectra of Ti-Co-Ce getter films at different depths in the as-deposited state and after activation at 400 °C, respectively. The as-deposited film rapidly oxidizes upon exposure to air, forming high-valent oxides on the surface metal alongside abundant oxygen-containing adsorbed species. As shown in Figure 7a, at room temperature, the O 1s signal maintains high intensity from the film surface to depths of approximately 10 nm, 20 nm, and 30 nm, indicating that the oxide layer and adsorbed oxygen are widely distributed throughout the shallow surface layer. After activation treatment involving holding at 400 °C for 0.5 h, the O 1s spectrum undergoes significant changes. As shown in Figure 7b, the O 1s signal intensity at various depths exhibits a marked decrease, indicating that heat treatment effectively reduces oxygen content near the surface. Furthermore, from the surface to depths of 10 nm, 20 nm, and 30 nm, the binding energy of the O 1s peak shifts gradually from the high-energy to the low-energy region. This reflects a systematic change in the chemical environment of oxygen with depth, further corroborating the migration and reduction behavior of oxygen from the surface to the interior during the heat treatment process.

Similarly, peak-by-peak fitting analysis was performed on the O 1s XPS spectrum. The fitting process employs a curve-fitting program based on iterative least-squares methods [28]. Two primary peaks were defined in the fitting process, constrained within the binding energy ranges of 529.5–530.5 eV (assigned to lattice oxygen in the metal oxide) and 530.5–531.5 eV (assigned to surface-adsorbed hydroxyl groups). During fitting, the FWHM of both components was maintained at approximately 1.5 ± 0.2 eV, consistent with typical values for oxide systems. Figure 7d–g and Figure 7h–k show the O 1s peak fitting spectra for the as-deposited state and the film surface after activation at 400 °C, respectively, at different etching depths (10 nm, 20 nm, 30 nm). By comparing the lattice oxygen peak areas obtained from the fitting, the trend of lattice oxygen content variation with depth before and after activation was determined. The statistical results are shown in Figure 7c. In the as-deposited state, the lattice oxygen content is highest at the film surface and gradually decreases with increasing depth (blue line in Figure 7c). After activation treatment at 400 °C, the lattice oxygen content at each depth was significantly lower than the corresponding values in the as-deposited state (orange lines in Figure 7c). The lattice oxygen content decreases sharply from the surface to approximately 10 nm. This phenomenon can be attributed to the combined effects of surface reduction reactions and inward diffusion of oxygen atoms. As the surface oxide layer is reduced, an oxygen concentration gradient forms from the surface to the interior, driving the released oxygen atoms to diffuse into the bulk phase and rapidly depleting the lattice oxygen content. As the etching depth increases, the decline in lattice oxygen gradually slows. At approximately 30 nm depth, it is only slightly lower than the pre-activation treatment state.

#### 3.3.4. Integrated Mechanism of Oxygen Diffusion and Reduction

Integrating the depth-profiled Ti 2p and O 1s spectra, a coherent model for oxygen dynamics during activation emerges. From the surface to a depth of approximately 10 nm, thermal activation provides energy, enabling a significant migration of lattice oxygen ions into the bulk phase. This region exhibits a high degree of oxidation prior to activation, resulting in a significant chemical potential gradient for oxygen diffusion into the bulk phase during activation. This creates a strong driving force for lattice oxygen diffusion, leading to a sharp decline in lattice oxygen content. As oxygen is lost, active elements such as titanium ions undergo reduction, and the oxidation state of titanium gradually decreases along the path Ti(IV) → Ti(III) → Ti(II). In the 20–30 nm region, reduction under thermal activation is accompanied by the inward diffusion of lattice oxygen from the surface. The degree of oxidation in the pre-activated getter film decreases from the surface toward the interior. Consequently, as depth increases, the oxygen chemisorption gradient gradually diminishes, and the driving force for lattice oxygen diffusion also decreases accordingly. In this region, lattice oxygen is introduced both through diffusion from the shallow surface layer and through its own generation via thermal activation reduction. At the same time, these lattice oxygen atoms continuously diffuse deeper into the bulk phase. Overall, the lattice oxygen content has only decreased slightly compared to before activation, remaining in a state of relative dynamic equilibrium. Moderate net oxygen loss drives partial reduction of titanium to its metallic state (Ti^0^). The relative enrichment of oxygen resulting from diffusion retardation provides the raw material for the formation of stoichiometric Ti_2_O_3_ (Ti(III)).

The 30 nm depth represents the critical threshold at which oxygen diffusion and reduction reactions reach dynamic equilibrium in Ti-Co-Ce getter films under thermal activation conditions at 400 °C. According to the Arrhenius equation, the position of this node is not fixed but is influenced by temperature and diffusion activation energy. To enable effective activation at lower temperatures, the key challenge lies in the innovative design of material composition and microstructure, so that complete oxygen diffusion can be achieved with a reduced thermal budget. Two primary strategies can be pursued: first, to systematically lower the activation energy (E_a_) for oxygen diffusion, thereby facilitating lattice oxygen migration; and second, to enhance energy utilization efficiency by concentrating thermal energy in regions where phase separation is most critical, thus promoting the necessary microstructural evolution at lower temperatures.

## 4. Conclusions

Ti-Co-Ce getter films were prepared via magnetron sputtering for microstructural analysis. To probe the activation mechanism critical for achieving low-temperature performance, we utilized in situ XPS with depth profiling, using monochromatic Al Kα radiation (hv = 1486.8 eV). This technique uniquely maps depth-resolved chemical transformations during the thermally driven removal of surface passivation. The main findings on the coupled oxygen reduction-diffusion dynamics are as follows:

(1) During thermal activation, the driving force for lattice oxygen diffusion into the bulk originates primarily from the chemical potential gradient, which diminishes with increasing depth. Simultaneously, the oxidation state of titanium undergoes a stepwise reduction from the surface to the interior (Ti(IV) → Ti(III) → Ti(II)), with the degree of reduction directly correlating to the net oxygen loss.

(2) During the thermal activation of Ti-Co-Ce getter films at 400 °C, lattice oxygen migrates extensively from the near-surface layer, causing a sharp decrease in lattice oxygen content in this region. At depths of 20–30 nm, moderate reduction coupled with relative oxygen enrichment jointly triggers phase separation. At approximately 30 nm depth, oxygen inflow and outflow reach a dynamic equilibrium.

(3) The position of the dynamic equilibrium node at approximately 30 nm is jointly controlled by temperature and the diffusion activation energy (E_a_). Therefore, to activate getter films at lower temperatures, the core strategy involves innovative material design through modifications in composition and microstructure. This approach systematically reduces the activation energy (E_a_) for oxygen diffusion. It maximizes energy utilization efficiency, ensuring that effective oxygen diffusion processes can still be driven and completed within a lower thermal budget.

## Data Availability

The original contributions presented in the study are included in the article. Further inquiries can be directed to the corresponding author.
